# Performance characteristics of a novel blood bag in-line closure device and subsequent product quality assessment

**DOI:** 10.1111/j.1537-2995.2010.02709.x

**Published:** 2010-10

**Authors:** Katherine Serrano, Elena Levin, Brankica Culibrk, Sandra Weiss, Ken Scammell, Wolfgang F Boecker, Dana V Devine

**Affiliations:** From Research and Development, Canadian Blood Services, and The University of British Columbia Centre for Blood ResearchVancouver, British Columbia, Canada; and Kabi Innovation Center, Fresenius Kabi Deutschland GmbHOberursel, Germany

## Abstract

**BACKGROUND:**

In high-volume processing environments, manual breakage of in-line closures can result in repetitive strain injury (RSI). Furthermore, these closures may be incorrectly opened causing shear-induced hemolysis. To overcome the variability of in-line closure use and minimize RSI, Fresenius Kabi developed a new in-line closure, the CompoFlow, with mechanical openers.

**STUDY DESIGN AND METHODS:**

The consistency of the performance of the CompoFlow closure device was assessed, as was its effect on component quality. A total of 188 RBC units using CompoFlow blood bag systems and 43 using the standard bag systems were produced using the buffy coat manufacturing method. Twenty-six CompoFlow platelet (PLT) concentrates and 10 control concentrates were prepared from pools of four buffy coats. RBCs were assessed on Days 1, 21, and 42 for cellular variables and hemolysis. PLTs were assessed on Days 1, 3, and 7 for morphology, CD62P expression, glucose, lactate, and pH. A total of 308 closures were excised after processing and the apertures were measured using digital image analysis.

**RESULTS:**

The use of the CompoFlow device significantly improved the mean extraction time with 0.46 ± 0.11 sec/mL for the CompoFlow units and 0.52 ± 0.13 sec/mL for the control units. The CompoFlow closures showed a highly reproducible aperture after opening (coefficient of variation, 15%) and the device always remained opened. PLT and RBC products showed acceptable storage variables with no differences between CompoFlow and control.

**CONCLUSIONS:**

The CompoFlow closure devices improved the level of process control and processing time of blood component production with no negative effects on product quality.

Many manual steps are performed by qualified staff during the processing of whole blood collections for blood component preparation. Some of these manual processing steps, for example, those involving a pinch grip posture, can lead to repetitive strain injuries (RSIs).^1^ In particular, the opening of in-line breakaway closures, which involves repeated gripping and pinching forces, can lead to RSI. In addition, the current design of breakaway closures involves the fracture of a hard plastic device and the manual separation of the pieces to facilitate appropriate blood flow. This may be inconsistently done, leading to excessive shear stress on the red blood cells (RBCs) and associated hemolysis.^2^ A new design of in-line closures developed by Fresenius Kabi (Bad Homburg, Germany) addresses the issues of both RSI and unanticipated hemolysis through a change in the mechanism by which the closures are opened. The new closure type consists of a nonfrangible plastic plug in the tubing; a focused lateral force causes the plug to deform and collapse on itself thereby opening a passage for the flow of blood components. In this study, the lateral pressure was applied by a manual hand-held device; however, in its final form the hand-held opener is electronic and operated by the push of a button. It is also possible to initiate the lateral force necessary to open the plug using an automated extractor (Compomat G5, Fresenius Kabi). This new closure design may also achieve more consistent opening of in-line closures than current breakaway in-line closure designs.

The aim of this study was to understand whether the configuration of the new closure device had any effect on blood component quality. Since shear stress can affect platelet (PLT) activation levels^3^ and can result in RBC damage,^4^,^5^ investigations focused on the quality of the cellular blood components produced using the in-line closures. The consistency of the performance of the closure device was also assessed.

## MATERIALS AND METHODS

### Blood component production

Blood was collected and processed according to standard operating procedures at Canadian Blood Services' development laboratory. All blood donors provided signed informed consent to participate in the study. Donors participating in the study were deferred from the regional blood donor pool for reasons other than their own health (e.g., travel deferral). The study was approved by Canadian Blood Services Research Ethics Board.

Whole blood units were collected using a quadruple blood bag system (CompoFlow, T&B 450/450/450/450 mL with in-line RBC filter and Composampler V, Fresenius Kabi). For comparison, whole blood units were prepared using a standard Fresenius Kabi blood bag system with the same configuration except for having traditional breakaway closures rather than CompoFlow closures. Whole blood was stored on cooling trays overnight. All units were processed for buffy coats using an automatic extractor (Compomat G4, Fresenius Kabi) and the settings recommended by the manufacturer. Extraction times were obtained from the electronic record of extraction for each unit. CompoFlow closures were opened, using a purpose-built hand-held prototype tool. RBC filtration times were obtained manually using timers. PLT pools of four buffy coats were made by the “train method.” Since CompoFlow closures are not symmetrical, the docking practice for the train was performed in a manner that placed like ends of the buffy coat bags adjacent to each other, that is, top to top or bottom to bottom. Thus, directional flow would occur either into the narrower opening first, through the wider side, into the next wide side, and out the second narrow opening or vice versa. All products were stored for the standard period under routine blood bank storage conditions. PLT concentrates (PCs) were stored at 22°C in a temperature-controlled incubator with agitation on a flatbed agitator (Helmer, Noblesville, IN). RBCs were stored in a 4°C refrigerator for 42 days. Plasma was examined immediately after preparation for signs of visual hemolysis and then frozen at −40°C. At the end of storage, cellular products were assessed for sterility to ensure that no contamination was introduced as a result of handling.

### Closure evaluation

**Fig. 1:**
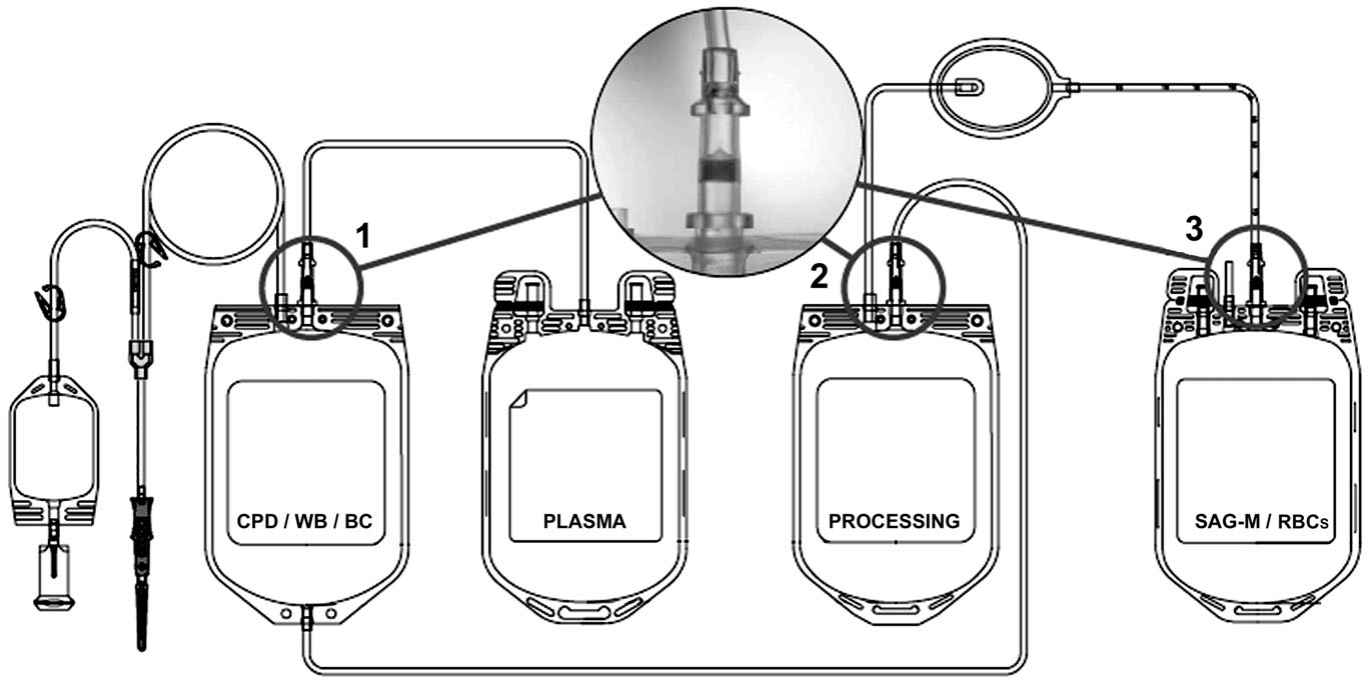
Schematic drawing of CompoFlow bag set depicting locations of the in-line closures: Closure 1 on the CPD/WB/BC bag, Closure 2 on the processing bag, and Closure 3 on the SAG-M/RBC bag. CPD = citrate-phosphate-dextrose; WB = whole blood; BC = buffy coat; SAG-M = saline-adenine-glucose-mannitol.

Each CompoFlow blood bag system has three in-line closures (Fig. 1). All three of the opened closures were excised from the line for aperture assessment. Closures were gently rinsed in saline followed by distilled water and let dry prior to image analysis. Closures 1 and 2 were generally assessed within 48 hours of opening, but Closure 3 was not assessed until after Day 42 since it could not be removed without compromising the RBC storage bag. Aperture images were obtained with a digital camera (QImaging Retiga 1300, Surrey, British Columbia, Canada) attached to a macroscope (Wild-Leitz Apozoom, Heerbrugg, Switzerland). Closures were positioned on a stage such that the camera's point of view was directed down the lumen of the tubing. Magnification was kept constant (zoom, 6.25). An internal standard consisting of a blunt-ended needle tip was placed in each image to ensure that the calibration was maintained. The mean pixel count of the needle width was 99, ranging from 96 to 102, in all images. Image analysis was conducted using computer software (Adobe PhotoShop, Adobe Systems, Inc., San Jose, CA) in which an area was delineated that represented the minimum aperture of each opened closure. Pixel counts were generated to provide quantitation of the aperture area. The method was assessed for reproducibility by measuring the same aperture 10 times with new placement of the closure on the macroscope stand each time; there was a high degree of reproducibility, with a coefficient of variance (CV) for the aperture area of 0.1%.

### RBC evaluation

A sample from the RBCs before filtration was collected using a sampling device (VFE0000Y, Macopharma, Mouvaux, France) attached by sterile docking. Completed RBCs were sampled aseptically using the ports on the bags into ethylenediaminetetraacetic acid (EDTA) tubes (Vacutainer, BD, Franklin Lakes, NJ) on Days 1, 21, and 42. A hematology analyzer (Advia 120, Siemens AG, Munich, Germany) was used to measure cell count and mean corpuscle volume (MCV). To obtain the total hemoglobin (Hb) measurement, RBCs were diluted 1/1000 with Drabkin's reagent (Sigma, St Louis, MO). For supernatant Hb, RBCs were subjected to a double centrifugation (2000 × *g* for 10 min and resulting supernatant at 15,000 × *g* for 15 min). The supernatant was diluted 1/5 with Drabkin's reagent. Samples were read using a plate reader (SpectraMax 190, Molecular Devices, Sunnyvale, CA) at 540 nm. A five-point calibration curve was created for each plate using a cyanmethemoglobin standard (Stanbio Laboratory, Boerne, TX). Hematocrit (Hct) was determined by collecting samples of RBCs in heparinized microcapillary tubes (Fisher Scientific, Nepean, Ontario, Canada), spinning in a microhematocrit centrifuge (IEC MB, Damon/IEC Division, Needham Heights, MA) for 5 minutes and visually quantifying the percentage of RBCs using a microcapillary reader (Damon/IEC Division). Total Hb measurements were made with Drabkin's assay and Hct measurements were made using microcapillary centrifugation because it has recently been shown that these assays can give more reliable measures of these variables than a hematology analyzer.^6^ Hemolysis was calculated according to the formula


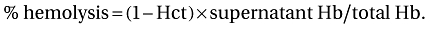


For a subset of randomly selected RBC units collected with the CompoFlow bag set, on Days 1, 7, 14, 21, 35, and 42, blood gas metabolic measurements at 37°C (pH, glucose, and lactate) were obtained using a blood gas analyzer (GEM Premier 3000, Instrumentation Laboratory, Orangeburg, NY). For results exceeding the normal range a 1:2 dilution in phosphate-buffered saline (PBS) of the sample was made and the test was repeated. With the exception of three samples, measurements occurred within 10 minutes of dilution. For samples with pH lower than detection level, measurements were performed using a pH meter (benchtop model 8005, VWR International, Plainfield, NJ) at room temperature. pH measurements were converted to pH(37°C) using the pH temperature correction equation recommended by Ashwood and coworkers.^7^

### PLT evaluation

PLT ability to swirl was assessed against direct light. A four-point scoring system was used: maximum score was given to the units with a defined swirl and minimum score to units with no swirl. All swirling assessments were performed by the same individual to minimize reader variability. PCs were sampled aseptically using the ports on the bags on Days 1, 3, and 7 of storage. PLT counts and mean PLT volume were obtained after a 10-minute incubation of the sample in an EDTA Vacutainer tube using the hematology analyzer. PLT activation of this sample was detected using a flow cytometer (FACSCanto II, BD Biosciences, San Jose, CA). PLTs were stained with fluorescent CD42a and CD62P antibodies (Immunotech, Marseille, France). Fluorescent isotype antibodies were used for the negative control. The PLT population was defined based on forward scatter and side scatter characteristics. Events with higher fluorescence than negative control were considered to be positive. Five-thousand events were collected in duplicate for each sample. A PC sample was also collected into a syringe for blood gas measurements at 37°C (pH, glucose, and lactate) and morphology scoring. For blood gas measurements exceeding the normal range, a 1:2 dilution in PBS of the sample was made and the test was repeated. Samples were measured within 10 minutes of dilution. PLT morphology was assessed by modified Kunicki morphology scoring.^8^ The PC sample was fixed with an equal volume of 4% paraformaldehyde. Samples were stored at room temperature and analyzed within 1 week by phase contrast microscopy at 1000× magnification. One-hundred PLTs were counted and categorized by shape as discoid, spiny sphere, or balloons. The morphology score was calculated by multiplying the number of discoid PLTs by 4, spiny spheres by 2, and balloons by 1 and then adding them together.

### Plasma evaluation for residual RBCs

Plasma units that appeared slightly pink were assessed for RBC content with a residual RBC (rRBC) enumeration assay developed by Canadian Blood Services using the Advia 120 CSF program (Siemens, Tarrytown, NY).^9^ Plasma was diluted 10 times in PBS and mixed with Advia 120 CSF reagent in a ratio of 1:1. After a 5- to 20-minute incubation at room temperature and thorough mixing, samples were analyzed using the Advia 120 CSF program mode.

### Residual white blood cell count

For RBCs and PCs, the presence of residual white blood cells (rWBCs) was detected with a commercially available kit (Leukocount, BD Biosciences). Briefly, 100 µL of sample was added to a tube containing a known amount of beads (Trucount, BD Biosciences). Leukocount reagent (400 µL) was added to the agitated mixture and incubated for 15 minutes in the dark. Flow cytometric analysis was performed on 10,000 events. The absolute rWBC count was calculated according to the manufacturer's instructions.

### Statistical analysis

A comparison of the blood components produced using CompoFlow bags versus standard bags was made at the end of the study. t test analyses (both paired and unpaired) were performed with computer software (Microsoft Excel, Microsoft Corp., Redmond, WA; or Prism 3.0, GraphPad, La Jolla, CA). Correlation analyses were performed with Microsoft Excel. Analysis of variance (ANOVA) and corresponding pairwise comparison posttests were performed using an online tool for statistical computation (VassarStats Website for Statistical Computation, http://faculty.vassar.edu/lowry/VassarStats.html). A p value of less than 0.05 was accepted as indicating significance.

## RESULTS

A total of 188 blood collections were made with CompoFlow bag sets on 12 separate days and 43 collections on 3 separate days were made with the control bag sets. Forty PCs were produced from CompoFlow collections and 10 PCs were produced from control collections.

Blood component volumes are summarized in Table 1. CompoFlow had slightly lower buffy coat volumes (p < 0.05, two-tailed t test) but at most, the true difference between the means is tiny and uninteresting. All other blood component volumes were not different between CompoFlow and control.

**TABLE 1:** Blood component volumes (mL)*

Bag system	Whole blood	RBC	Plasma	Buffy coat†	Pooled PLTs
CompoFlow‡	451 ± 19 (245-543, 451)	256 ± 19 (151-316, 256)	281 ± 20 (180-336, 283)	50 ± 2 (44-58, 50)	329 ± 11 (301-351, 326)
Control§	444 ± 23 (300-453, 450)	253 ± 16 (195-268, 254)	278 ± 20 (189-308, 278)	51 ± 3 (46-59, 50)	331 ± 8 (321-345, 330)

* Data are expressed as mean ± 1 SD (range, median).

† p = 0.01, CompoFlow versus control.

‡ Whole blood, RBCs, plasma, and buffy coat, n = 188; pooled PLTs, n = 40.

§ Whole blood, RBCs, plasma, and buffy coat, n = 43; pooled PLTs, n = 10.

### CompoFlow closures

**Fig. 2:**
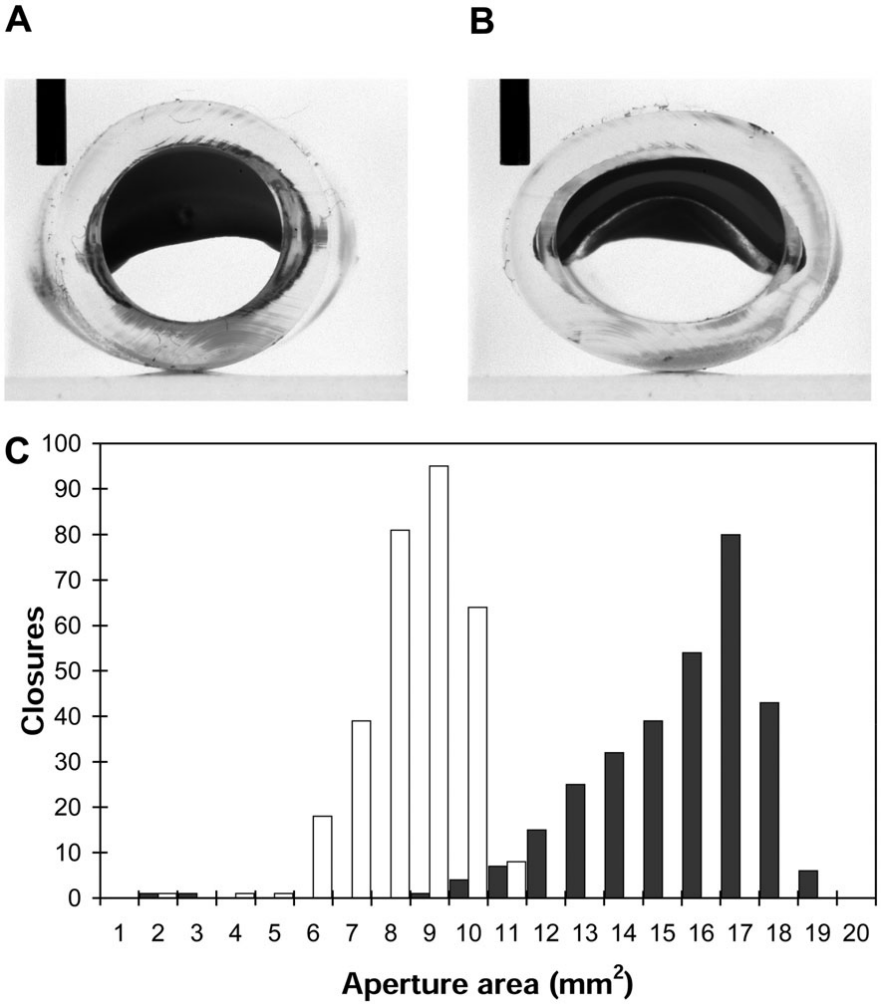
CompoFlow image analysis. Representative images of opened CompoFlow closures looking down the lumen of the tubing at the pointed end of the closure (A) and the bottom end of the closure (B). The black bar at the top left corner of each image is the internal standard (width, 0.9 mm). Frequency histogram of closure aperture area at the pointed end (□) and the bottom end (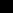; C).

In total, 308 CompoFlow closures were excised from their respective bags for analysis after blood component production: 102 closures from the buffy coat bag (Closure 1), 101 closures from the processing bag (Closure 2), and 105 closures from the RBC storage bag (Closure 3). In no case was a closure found to return to its original state after opening, irrespective of elapsed time between opening and analysis (1 to >42 days); once opened, the opening is permanent. Images of closures were taken from both sides of each closure (Figs. 2A and 2B). The closure openings were smaller at the “pointed” end (8.0 ± 1.2 mm^2^; CV, 15%) than at the “bottom” end (15.0 ± 2.3 mm^2^; CV, 15%) with a slightly skewed distribution for the opening area at the bottom end (Fig. 2C). The area of the aperture at the pointed end was approximately 38% that of the cross-sectional area of the tubing (21 ± 0.2 mm^2^). Correlation statistics were therefore performed using the data from the pointed end because this was felt to be the most rigorous challenge for the cells in terms of passing through the aperture.

### Extraction and filtration

The time required for extraction of the different components on the Compomat G4 was monitored to determine whether the CompoFlow closures affected the flow rate of the extraction. Extraction time was normalized against the volume of the whole blood units. Units for which the tubing was kinked during extraction or for which a manual stop of the extraction process was required were not included in the analysis. Similarly the time required for RBC filtration was monitored and filtration time was normalized against the volume of the RBCs. Units for which filters were not primed in the correct orientation were not included in the filtration time analysis. Extraction time was significantly shorter with the CompoFlow bag sets (p = 0.01, two-tailed t test). There was no difference in RBC filtration time detected between CompoFlow and control bag sets (Table 2). There was no correlation between the combined CompoFlow closure aperture area of Closures 1 and 2 and the total extraction time (r = 0.03, n = 93). The CompoFlow closures do not appear to limit the flow during extraction.

**TABLE 2:** Normalized extraction and RBC filtration times (sec/mL)*

Bag system	Extraction time†	RBC filtration time
CompoFlow‡	0.46 ± 0.11 (0.34-1.48, 0.44)	8.1 ± 1.3 (4.0-13.4, 8.2)
Control§	0.52 ± 0.13 (0.42-1.12, 0.47)	7.8 ± 1.1 (5.8-12.5, 7.6)

* Data are expressed as mean ± 1 SD (range, median).

† p = 0.01, CompoFlow versus control.

‡ Extraction time, n = 178; RBC filtration time, n = 121.

§ Extraction time, n = 42; RBC filtration time, n = 43.

### RBCs

Due to work scheduling, 23 RBC units were sampled on Day 22 instead of Day 21 and 32 RBC units were sampled on Day 43 instead of Day 42; the data from these units were pooled with Day 21 and Day 42 data sets, respectively. The first batch of prefiltration samples suffered a sampling error; therefore, these data were omitted from all analyses. RBC count, Hb levels, and Hct decreased from the prefiltration sample to the postfiltration sample (p < 0.01), indicating that there was RBC loss occurring during the filtration process as might be expected. RBC count, Hb levels, and Hct stayed fairly consistent thereafter (Table 3). This trend was similar for both CompoFlow and control samples and there was no difference between the two bag systems for either RBC count or total Hb. The Hct of the CompoFlow group was slightly higher than that of the control group (p < 0.01, two-factor ANOVA with repeated measures on one factor), although this is not clinically meaningful and statistical significance was only achieved because the data set had limited variability. A different trend was noticed for RBC MCV (Table 3). As expected the MCV was not different before and after RBC filtration. However, upon storage the MCV increased for both CompoFlow and control samples (p < 0.01); this is consistent with RBC swelling and could be due to inefficient operation of the Na^+^/K^+^ pumps in cold storage.

**TABLE 3:** RBC variables*

		After filtration		
		
Variable	Before filtration, Day 1	Day 1	Day 21	Day 42
RBC count (×10^12^/L)				
CompoFlow†	6.9 ± 0.4 (5.1-8.6, 6.9)	6.1 ± 0.4 (4.0-6.9, 6.1)	6.2 ± 0.5 (4.1-7.9, 6.2)	6.1 ± 0.5 (4.0-8.2, 6.1)
Control‡	6.9 ± 0.3 (6.3-7.8, 7.0)	6.1 ± 0.4 (5.4-6.9, 6.2)	6.0 ± 0.4 (5.0-6.9, 5.9)	6.0 ± 0.4 (5.3-6.8, 6.0)
Hb (g/L)				
CompoFlow	215 ± 11 (158-253, 216)	191 ± 11 (127-214, 192)	195 ± 17 (126-257, 193)	192 ± 17 (127-258, 191)
Control	217 ± 8 (195-234, 218)	191 ± 9 (167-206, 192)	189 ± 14 (159-235, 188)	189 ± 10 (164-219, 189)
Hct (L/L)				
CompoFlow	0.67 ± 0.03 (0.49-0.80, 0.68)	0.60 ± 0.03 (0.39-0.66, 0.60)	0.60 ± 0.05 (0.39-0.80, 0.60)	0.60 ± 0.05 (0.39-0.79, 0.59)
Control	0.67 ± 0.02 (0.60-0.72, 0.68)	0.59 ± 0.02 (0.50-0.63, 0.60)	0.58 ± 0.04 (0.49-0.68, 0.58)	0.58 ± 0.03 (0.50-0.64, 0.59)
MCV (fL)				
CompoFlow	97 ± 4 (87-107, 97)	98 ± 4 (87-107, 98)	100 ± 4 (89-110, 100)	102 ± 4 (90-112, 102)
Control	95 ± 4 (86-104, 96)	97 ± 4 (85-105, 97)	99 ± 4 (87-106, 100)	101 ± 5 (88-110, 101)

* Data are expressed as mean ± 1 SD (range, median).

† CompoFlow before filtration Day 1, n = 172; after filtration Day 1, Day 21, and Day 42, n = 188.

‡ Control before filtration Day 1, n = 42; after filtration Day 1, Day 21, and Day 42, n = 43.

**Fig. 3:**
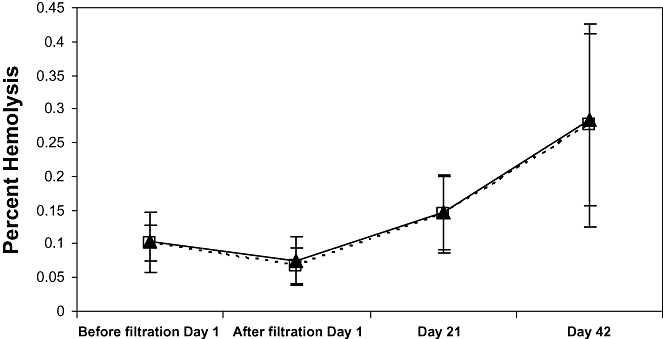
Mean percent RBC hemolysis of RBCs on Day 1 (before and after filtration), Day 21, and Day 42 for CompoFlow (▴, —) and control (□, - - -). CompoFlow, n = 188, except before filtration Day 1, n = 171; control, n = 43.

RBC hemolysis measurements decreased slightly after the filtration step (Fig. 3), suggesting some loss of supernatant Hb on the filter. Percent hemolysis increased significantly (p < 0.01) over storage but there was no difference in percent hemolysis between CompoFlow and control samples (two-factor ANOVA with repeated measures on one factor). It appears that the CompoFlow closures do not negatively impact RBC integrity any more than a correctly opened traditional breakaway closure might. The European Council guidelines state that hemolysis must be less than 0.8% of RBC mass^10^ and FDA guidance suggests that there be no more than 1% hemolysis at the end of the storage period.^11^ One CompoFlow RBC unit and one control RBC unit had hemolysis levels greater than this with 1.086 and 0.984% hemolysis, respectively. The relationship between percent hemolysis and the size of the CompoFlow aperture was analyzed for the four RBC hemolysis measurements (Day 1 before filtration, Day 1 after filtration, Day 21, and Day 42) versus the aperture sizes of the CompoFlow closures from the processing bag (Closure 2) and the final RBC storage bag (Closure 3). There was no correlation between the percent hemolysis measurements and the CompoFlow apertures for either of the opened closures that the RBC pass through during production.

Blood gas/electrolyte measurements for a random subset of 10 CompoFlow RBC units are reported in Table 4. pH decreased from Day 1 to Day 14 (p < 0.01) and remained constant for the remainder of the storage period whereas pO_2_ slowly increased over the storage period (p < 0.001). Glucose levels decreased from Day 1 to Day 14 and from Day 14 to Day 28 (p < 0.01). While not attaining significance after Day 28, the glucose levels appeared to slowly continue their downward trend to Day 42. Complementing the glucose measurements, lactate levels increased from Day 1 to Day 14 and from Day 14 to Day 28 (p < 0.01, one-way ANOVA for five correlated samples), appearing to slowly continue the upward trend to Day 42, although again not significant past Day 28. rWBC count for these units ranged from 6360 to 199,538 rWBCs/unit, averaging 69,483 ± 57,587 rWBCs/unit. This subset of RBCs met AABB standards for rWBCs in leukoreduced RBCs (<5 × 10^6^ WBCs/unit)^12^ as well as Council of Europe standards (<1 × 10^6^ WBCs/unit).^10^

**TABLE 4:** RBC blood gas/electrolytes*

Variable	Day 1	Day 14	Day 28	Day 35	Day 42
pH	6.75 ± 0.10 (6.55-6.88, 6.76)	6.41 ± 0.09 (6.17-6.51, 6.43)	6.32 ± 0.15 (6.16-6.56, 6.28)	6.36 ± 0.13 (6.10-6.52, 6.39)	6.35 ± 0.07 (6.25-6.44, 6.33)
pO_2_ (mmHg)	32 ± 11 (21-51, 28)	45 ± 14 (33-72, 38)	65 ± 27 (43-123, 54)	76 ± 29 (51-141, 62)	86 ± 38 (52-164, 71)
Glucose (mmol/L)	29 ± 4 (23-35, 29)	24 ± 2 (19-27, 24)	19 ± 2 (15-21, 20)	18 ± 2 (13-20, 19)	17 ± 2 (11-19, 18)
Lactate (mmol/L)	6 ± 1 (4-7, 5)	13 ± 2 (10-17, 13)	17 ± 2 (14-20, 17)	19 ± 4 (15-26, 18)	21 ± 4 (16-27, 19)

* Data are reported as mean ± 1 SD (range, median), n = 10.

### PLTs

The units prepared during the first 4 collection days did not use the final centrifuge packing scheme and it was deemed that due to the high sensitivity of PLTs to shear stress, this may lead to a disturbance of the PLTs. The PCs from these first four collections were therefore omitted from the PLT analysis. Of 26 CompoFlow PCs, two PCs contained PLTs originating from donors who had ingested acetylsalicylic acid (in one of four buffy coats in the pool), one PC was made with two slightly low-volume buffy coats (44 mL each), and one PC exceeded the 28-hour processing time by 10 minutes. These units were included in the final analysis. Of these, 4 units were not sampled on Day 3 for reasons of logistics.

Measurements of PC quality variables are listed in Table 5. While PLT surface expression of CD62P increased significantly (p < 0.01) with time of storage reflecting the development of the PLT storage lesion, there was no difference detected between CompoFlow and control PCs. CD62P expression data were analyzed together with the size of the aperture of the buffy coat closure openings (Closure 1) to determine whether a correlation between these measurements might exist. It was hypothesized that a smaller aperture size might result in increased PLT activation. Since the PLTs pass through one or three of these buffy coat closure openings as the train is pooled into one larger unit, the correlation was determined for both the mean and the minimum aperture areas of these three closures. There was no correlation between CD62P expression and the CompoFlow opening mean aperture or between CD62P expression and the CompoFlow opening minimum aperture (n = 22). Morphology scores decreased significantly over the storage period as expected (p < 0.01) and there was no difference between CompoFlow or control PCs. As for CD62P expression, there was no correlation between morphology and the CompoFlow aperture area. Visual swirl, which like morphology scoring, assesses PLT shape change, also decreased over time (p < 0.01). PC pH followed a characteristic trend of increasing to Day 3 and then decreasing to Day 7. The differences between days were significant (p < 0.01). All units met the Canadian standards quality control testing requirement that the pH remain within the range 6.4 to 7.4. Glucose decreased significantly over the days of storage and, correspondingly, lactate concentrations increased significantly throughout storage (p < 0.01). For all of the PLT quality variables measured, there was no measurable difference between CompoFlow and control PCs (multifactorial ANOVA and Tukey's “honestly significant difference” pairwise comparisons test).

**TABLE 5:** PLT quality variables on Days 1, 3, and 7 of storage*

	CompoFlow†			Control‡		
		
Variable	Day 1	Day 3	Day 7	Day 1	Day 3	Day 7
PLTs/unit (×10^9^)	300 ± 49 (235-397, 288)	269 ± 46 (199-369, 263)	286 ± 52 (204-381, 289)	293 ± 51 (215-389, 295)	273 ± 50 (190-342, 282)	276 ± 51 (199-354, 284)
MPV (fL)	7.7 ± 0.5 (7.1-8.9, 7.7)	8.4 ± 0.4 (7.7-9.2, 8.4)	8.3 ± 0.4 (7.6-9.1, 8.3)	7.9 ± 0.4 (7.2-8.4, 8.0)	8.5 ± 0.4 (7.9-9.4, 8.4)	8.3 ± 0.3 (7.8-8.8, 8.2)
%CD62P expression	16 ± 6 (6.3-34.2, 17.4)	18 ± 5 (9.3-27.2, 17.8)	33 ± 7 (18.5-44.0, 32.4)	16 ± 4 (10.1-19.7, 17.0)	21 ± 6 (13.3-34.5, 20.1)	34 ± 6 (27.4-46.1, 32.9)
CD62P mean fluorescence	1021 ± 269 (664-1932, 1019)	1549 ± 357 (988-2258, 1520)	2038 ± 453 (1347-3291, 2076)	957 ± 195 (738-1215, 888)	1967 ± 612 (1228-2951, 1759)	2162 ± 392 (1851-3110, 1971)
Morphology	348 ± 15 (317-368, 351)	328 ± 12 (299-341, 329)	292 ± 19 (266-337, 291)	352 ± 9 (341-370, 348)	327 ± 22 (293-357, 328)	297 ± 18 (277-329, 299)
Swirl	4.0 ± 0.1 (3.5-4.0, 4.0)	3.5 ± 0.3 (3.0-4.0, 3.5)	2.8 ± 0.3 (2.5-3.0, 3.0)	4.0 ± 0.0 (4.0-4.0, 4.0)	3.4 ± 0.3 (2.5-3.5, 3.5)	2.9 ± 0.5 (1.5-3.0, 3.0)
pH	7.08 ± 0.03 (7.00-7.14, 7.08)	7.32 ± 0.03 (7.28-7.37, 7.32)	7.25 ± 0.05 (7.14-7.36, 7.26)	7.08 ± 0.02 (7.04-7.12, 7.08)	7.32 ± 0.03 (7.27-7.36, 7.32)	7.24 ± 0.05 (7.17-7.33, 7.23)
Glucose (mmol/L)	18.8 ± 1.3 (16.7-22.5, 18.9)	17.1 ± 0.9 (15.3-18.8, 17.0)	14.6 ± 1.1 (12.4-16.5, 14.4)	18.3 ± 0.9 (17.0-19.3, 18.5)	16.6 ± 0.8 (15.2-17.6, 16.9)	13.8 ± 1.2 (11.9-15.9, 13.9)
Lactate (mmol/L)	7.9 ± 0.8 (6.1-10.5, 7.9)	9.6 ± 0.7 (7.9-10.6, 9.7)	14.8 ± 1.0 (13.6-17.4, 14.5)	7.7 ± 0.6 (6.8-8.5, 7.7)	9.4 ± 0.5 (8.9-10.4, 9.3)	14.5 ± 0.7 (13.0-15.6, 14.6)

* Data are expressed as mean ± 1 SD (range, median).

† CompoFlow Day 1 and Day 7, n = 26; Day 3, n = 22.

‡ Control, n = 10.

A randomly selected subset (n = 10) of the CompoFlow PC units was examined for rWBC content. It was expected that the amount of WBC contamination in the units would be low since all units were subjected to leukoreduction via filtration. The rWBC count on Day 1 was 5805 ± 5450 WBCs/unit with a range of 0 to 15,623 WBCs/unit. This subset of PCs met AABB standards for rWBCs in pooled, leukoreduced PCs (<5 × 10^6^ WBCs)^12^ as well as Council of Europe standards (<0.2 × 10^6^ WBCs/single-unit equivalent).^10^

### Plasma

Seven “pink” plasma units were selected for measurement of RBC contamination (rRBCs) and Hb content. These plasma units did have RBCs settling out in the tubing, but with the exception of possibly 1 unit, these units were not sufficiently “red” to have been flagged in a routine production environment. rRBC content in these units ranged from 0.25 × 10^9^ to 10 × 10^9^ rRBCs/L (75 × 10^6^ to 2552 × 10^6^ rRBCs/unit) with a range of 0.37 to 1.48 g/L Hb. With the exception of 1 unit, these plasma units met the Council of Europe specifications for rRBCs (<6.0 × 10^9^/L).

None of the PCs or RBCs were contaminated with bacteria as determined using an 8-mL inoculum of sample in an aerobic bottle of the BacT/ALERT bacterial detection system immediately after completion of the storage period.

## DISCUSSION

The CompoFlow blood bag system introduces a new approach to in-line blood bag closures with a design that departs substantially from the currently used breakaway closures. In this study CompoFlow closures were opened by a hand-held mechanical prototype device, that applied pressure on the tubing external to the closure. Although not tested in this study, CompoFlow closures are designed to be opened with an automated device. In its final form for the marketplace, the device opener will be a hand-held electronic device or will be integral to the semiautomated extractor.

Digital image analysis indicated that the CompoFlow closures provided an aperture that displayed a fairly reproducible cross-sectional area (CV, 15%). This opening was similar in size whether measured within a couple of days of opening the in-line closure or whether measured after 6 weeks of opening. It must be noted that the cross-sectional area measured at the pointed end of the closure with our macroscope setup is only an approximation of the actual cross-sectional area. The reason for this is that the aperture outline for the images taken at the pointed end of the closure is drawn in a single plane; however, the outline in fact represents a line that travels in multiple planes. Restricted blood flow with the potential for disruption of cellular components is possible if a standard breakaway closure is improperly opened by not being broken completely. A design feature of the CompoFlow opening devices is that once pressure is initiated, the clamping motion must reach a threshold distance before the device will disengage from the tubing. In this way, partial opening of the CompoFlow closures is prevented.

In addition to the observation that CompoFlow closures do not appear to be limiting flow during extraction as determined through correlation statistics of extraction time versus aperture size, extraction times using the CompoFlow bag system were significantly shorter than those of standard bag sets. For a unit of 450 mL of whole blood the mean extraction time using CompoFlow closures would be 3 minutes 27 seconds whereas using standard breakaway closures the extraction time of a 450-mL whole blood unit would average 3 minutes 54 seconds. This corresponds to a decrease of approximately 12% in processing time for the extraction step. Over the course of a day in a large blood center, this difference has the potential to translate into a significant gain in production efficiency.

RBCs are subjected to various mechanical forces as well as nonmechanical stimuli during processing, which can lead to increased hemolysis. Some of these stimuli include incorrect anticoagulant ratios, passage through partially opened transfer tube closures, temperature fluctuations, turbulence with rapid mixing, passage past the edges of kinked tubing, resuspension of cell pellets, and passage through filters.^13^ In this study, passage of RBCs past CompoFlow closures did not appear to expose RBCs to increased damaging shear forces or turbulence as there was no difference in hemolysis between CompoFlow and control units. A small amount of RBC hemolysis did continue over time in both CompoFlow and control units as is known to occur in storage but on Day 42 both groups averaged less than 0.3% hemolysis, which is well within both the recommended American and the recommended European Guidelines.^11^

PLTs also respond to the forces and conditions they encounter during processing. PLTs become partially activated as determined by activation of GPIIbIIIa receptors and expression of the degranulation marker CD62P during those processing steps that occur after the initial phlebotomy of the blood.^14^ In the study by Metcalfe and colleagues,^14^ CD62P levels were found to be very informative because they were able to identify significant differences in PLT activation between different PLT production methods. Here we report no difference in CD62P between PLTs produced either with CompoFlow or with control bag systems either on the day of production or after storage for 3 and 7 days. Other PLT quality measures, pH, swirl, morphology, glucose, and lactate, similarly mirrored a lack of difference between CompoFlow and control. The apertures formed by the CompoFlow closures do not appear to cause areas of turbulence or shear stress that increase PLT activation compared to breakaway closures.

CompoFlow closures impart an ergonomic system for opening in-line closures which result in stable and reproducible apertures. Cellular products produced using CompoFlow bag systems do not appear different in quality from those produced using standard breakaway closures and they meet the requirements of American and European standard setting bodies.

## ABBREVIATIONS

PC(s): platelet concentrate(s)
rRBC(s): residual red blood cell(s)
RSI: repetitive strain injury
rWBC(s): residual white blood cell(s)

## References

[1] Keyserling WM (2000). Workplace risk factors and occupational musculoskeletal disorders, part 2: a review of biomechanical and psychophysical research on risk factors associated with upper extremity disorders. Am Ind Hyg Assoc J.

[2] Knels R, Geusendam G, Müeller-Kuller T, Sireis W (2008). Influence of blood bag breakables on the quality of blood products [abstract]. Vox Sang.

[3] Kroll MH, Hellums JD, McIntire LV, Schafer AI, Moake JL (1996). Platelets and shear stress. Blood.

[4] Duffy R, Tomashek K, Spangenberg M, Spry L, Dwyer D, Safranek TJ, Ying C, Portesi D, Divan H, Kobrenski J, Arduino M, Tokars J, Jarvis W (2000). Multistate outbreak of hemolysis in hemodialysis patients traced to faulty blood tubing sets. Kidney Int.

[5] Leverett LB, Hellums JD, Alfrey CP (1972). Red blood cell damage by shear stress. Biophys J.

[6] Han V, Serrano K, Devine DV (2010). A comparative study of common techniques used to measure haemolysis in stored red cell concentrates. Vox Sang.

[7] Ashwood ER, Kost G, Kenny M (1983). Temperature correction of blood-gas and pH measurements. Clin Chem.

[8] Kunicki TJ, Tuccelli M, Becker GA, Aster GH (1975). A study of variables affecting the quality of platelets stored at “room temperature. Transfusion.

[9] Culibrk B, Levin E, Weiss S, Devine DV (2006). Assessment of residual cell counts in blood products using the Bayer Advia 120 cerebrospinal fluid assay. Transfusion.

[10] Council of Europe (2007). Guide to the preparation, use and quality assurance of blood components.

[11] FDA summary basis of approval for red blood cells frozen and red blood cells deglycerolized (Reference number 86-0335) (1986).

[12] American Association of Blood Banks (2009). Standards for blood banks and transfusion services.

[13] Sowemimo-Coker SO (2002). Red blood cell hemolysis during processing. Transfus Med Rev.

[14] Metcalfe P, Williamson LM, Reutelingsperger CP, Swann I, Ouwehand WH, Goodall AH (1997). Activation during preparation of therapeutic platelets affects deterioration during storage: a comparative flow cytometric study of different production methods. Br J Haematol.

